# Blood Vitamin Concentrations in Pond Sliders (*Trachemys scripta*) Under Human Care in Central Europe and Possible Seasonal and Sex-Specific Influences

**DOI:** 10.3390/ani15060859

**Published:** 2025-03-17

**Authors:** Christoph Leineweber, Gregor Geisler, Sabine Öfner, Rachel E. Marschang

**Affiliations:** 1 Laboklin GmbH & Co. KG, Steubenstrasse 4, 97688 Bad Kissingen, Germany; 2 Reptile Rescue Center Munich e.V., Kaulbachstrasse 37, 80539 Munich, Germany

**Keywords:** reference interval, season, turtles, vitamin A, vitamin B, vitamin E

## Abstract

Vitamin A deficiency is common in pond sliders, and vitamin E and B1 deficiencies have also been described in carnivorous aquatic species such as crocodiles, but otherwise little is known about vitamin concentrations in the blood of reptiles, and reference intervals only exist for a few vitamins in a few species. The aim of this study was therefore to measure vitamins A, B1, B2, B6, B9, B12, and E in the heparinized whole blood and plasma of pond sliders (*Trachemys scripta*) (*n* = 188) from April to September 2022 using high-performance liquid chromatography (HPLC) and spectrophotometry. In addition to establishing reference intervals, possible influencing factors, including the season of blood sampling and the sex of the turtles, were evaluated. The median concentrations measured were as follows: vitamin A—0.17 mg/L, B1—59.10 µg/L, B2—693.50 µg/L, B6—2.375 µg/L, B9—16.06 ng/mL, B12—3225.00 pg/mL, and E—7.59 mg/L. Significant (*p* ≤ 0.05) seasonal variations were found for vitamin A, B1, and B9, and sex-specific variations were found for vitamin E. The results show that factors such as sex and the time of year play a role in vitamin levels in the blood of turtles and should be considered when interpreting blood results. The reference intervals established in the present study serve as a basis for further studies and help to control and improve the vitamin supply of the turtles, contributing to the better husbandry and nutrition of this species in human care.

## Simple Summary

Little is known about vitamin concentrations in the blood of reptiles and reference intervals are limited. The aim of this study was therefore to measure vitamins A, B1, B2, B6, B9, B12, and E in the heparinized whole blood and plasma of pond sliders (*Trachemys scripta*) (*n* = 188) from April to September 2022 using high-performance liquid chromatography (HPLC) and spectrophotometry, and to evaluate the influence of sex and season on the measured values. Significant (*p* ≤ 0.05) seasonal variations were found for vitamins A, B1, and B9, and sex-specific variations were found for vitamin E, indicating that all of these factors should be considered when establishing and interpreting reference intervals in turtles.

## 1. Introduction

Most vitamins have to be ingested with the diet and cannot be synthesized by the body. Carnivorous reptile species in particular are susceptible to vitamin deficiencies, especially if they are fed meat rather than whole prey [1]. These deficiencies can be clinically relevant. For example, retinol (vitamin A) deficiency leads to the hyperkeratosis of epithelial cells, which block the glandular ducts, and is commonly associated with swelling of the ear glands and otitis in aquatic turtles [1,2]. Thiamine pyrophosphate (vitamin B1) deficiency, which is mostly observed in piscivorous species, leads to neurological disorders like the loss of the righting reflex, torpor, depression, and incoordination due to possible bacterial thiaminases [1,3,4]. Tocopherol (vitamin E) deficiency has been documented to cause steatites, fat necrosis, and muscle degeneration in several reptile species, including crocodiles, snakes, lizards, and sea turtles [5,6,7,8,9,10,11]. Despite these documented cases of clinical disease associated with various vitamin deficiencies, little is known about physiological vitamin levels in reptiles and, above all, blood reference intervals to monitor the sufficient supply of these vitamins in these animals are limited and not available for all vitamins and reptile species [12,13,14,15,16,17].

Pond sliders (*Trachemys scripta*) were originally found from northern Mexico to the south–central and southeastern USA [18]. The three subspecies of this turtle species have different distribution areas. Red-eared slider turtles (*Trachemys scripta elegans*) live in the Mississippi river system from Illinois to the Gulf of Mexico [18]. Yellow-eared slider turtles (*Trachemys scripta scripta*) are common from southeast Virginia to northern Florida, and Cumberland slider turtles (*Trachemys scripta troostii*) from southwest Virginia and Kentucky to northeast Alabama [18]. Slider turtles were once commonly kept as pets, but the species are now invasive in many parts of the world, including Europe. These turtles are omnivorous, with the carnivorous proportion of the diet decreasing from juveniles to adults [19]. Due to their diet and sometimes unfavourable husbandry and feeding conditions, this species is frequently affected by vitamin deficiencies and presented in practice with classic signs such as otitis [1,2].

Due to the differences in diets between juveniles and adults, it is to be expected that there are differences in the blood vitamin concentrations between age groups. Many other blood analytes, such as minerals, are also known to vary between sexes and seasons in turtles [20,21,22,23].

The aim of this study was to establish blood reference intervals for adult clinically healthy pond sliders for vitamins A, B1, B2, B6, B9, B12, and E and to evaluate whether the blood levels of these vitamins differ between sexes and seasons.

## 2. Materials and Methods

### 2.1. Animals

Blood samples were collected from 188 clinically healthy adult pond sliders (53 males, 135 females) from April to September 2022. The samples were collected during the course of routine health checks. No samples were collected in fall or winter due to the hibernation of the turtles during these seasons. The use of portions of the samples left over after routine blood testing was approved by the ethics commission of the Faculty of Veterinary Medicine of the University of Leipzig (GZ: EK 21/2021). The turtles weighed between 400 g and 3500 g (x̄ = 1364 g), and classification of the animals as adults was based on their weights. Samples were grouped into seasons depending on when the sample was collected. April to May was considered spring (*n* = 7), June to July as early summer (*n* = 54), and August to September as late summer (*n* = 127). The turtles were kept in naturalistic ponds by private keepers, public zoological institutions, or reptile rescue centres in Germany, and were fed ad libitum with industrially manufactured turtle pellets (different brands), invertebrates, wild herbs, and water plants. The animals had been kept at each of these locations for varying lengths of time and the origins and history of many of the animals was not known. Only turtles with no history of disease in the past months and that were eating normally and were considered healthy were included in the study.

All turtles were considered healthy on the basis of a general health check at the time of blood collection. Health checks included evaluation of the shell, skin, and nails, as well as behaviour and physical strength. Animals sampled in the spring had all begun eating following hibernation. All of the animals sampled were considered in good body condition.

### 2.2. Sample Collection and Analysis

Blood samples were collected from the dorsal coccygeal vein. In animals in which this approach was not successful, blood was collected from the subcarapacial plexus. No samples with visible lymph contamination or a PCV < 10% were included in the study. The amount of blood collected depended on the weight of the turtle and ranged from 0.5 to 3.0 mL, never exceeding 0.8% of the total body weight [24]. Blood was collected in lithium-heparinized tubes (4.5 mL tube, lithium heparin, Sarstedt AG & Co KG, 51,588 Nurnbrecht, Germany) and transported in an upright position at cool temperatures (4–8 °C, 39.2–46.4 °F) overnight to the laboratory. Whole blood was used for the measurement of vitamins B1, flavin adenine dinucleotide (B2), and pyridoxal (B6). If there was still a sufficient amount of blood left over, the remaining whole blood was centrifuged at 3220× *g* for 3 min in a Thermo Scientific Megafuge ST Plus Series (Thermo Fisher Scientific Inc., Breda, The Netherlands) no later than 24 h after collection. The plasma was used to measure vitamin A, E, folic acid (B9), and cobalamin (B12) concentrations. Samples were kept cool in storage (8 °C; 46.4 °F) and protected from light until testing. Due to the limited blood volume available from some of the turtles, it was not possible to measure all vitamins in all samples (Table 1). Vitamins A and E were measured from heparinized plasma, and B1, B2, and B6 were measured from heparinized whole blood by high-performance liquid chromatography (HPLC; Prominence HPLC System, Shimadzu Deutschland GmbH, Duisburg, Germany) using RECIPE ClinRep^®^ Complete Kits (RECIPE Chemicals + Instruments GmbH, 80,992 Munich, Germany). Vitamins B9 and B12 were measured from heparinized plasma spectrophotometrically using the cobas^®^ 8000 analyser module e602 (Roche Diagnostics, Mannheim, Germany). The concentrations of individual vitamins in some of the samples were below the limit of quantification (LOQ) of the tests, and in these cases half of the quantification limit was used for statistical analysis and the calculation of reference intervals [25]. The LOQs for each test and the number of samples that fell below that limit were as follows: B1 (LOQ 1 µg/L; *n* = 1), B6 (LOQ 0.4 µg/L; *n* = 3), and E (LOQ 0.2 mg/L; *n* = 5). For vitamins B9 and B12, some samples were above the LOQ and were diluted with physiological saline solution and then measured again (B9 >20 ng/mL; *n* = 52 and B12 > 2000 pg/mL; *n* = 121). All assays were validated according to the Clinical and Laboratory Standards Institute (CLSI) guidelines and the inter- and intra-assay coefficients of variability were within the recommended limits.

### 2.3. Statistical Analyses

Statistical analyses were carried out using the SAS analysis software (SAS OnDemand for academics; SAS Institute Inc., Cary, NC, USA), using the Anderson–Darling test for determinations of normality. An ANOVA mixed model was used to evaluate possible seasonal and sex-specific influences and their interactions, with an alpha of *p* < 0.05 considered significant. Reference intervals were determined according to the guidelines of the American Society of Veterinary Clinical Pathologists (ASVCP) [26] using the Reference Value Advisor v2.1 and the nonparametric method [27], with the exception of the values for spring due to the low number of samples (*n* = 7). Outliers were determined using Tukey and Dixon–Reed tests, but were not excluded from the statistical analyses.

## 3. Results

The calculated reference intervals for each vitamin in pond sliders are shown in Table 1 and Table 2. Due to the low number of samples collected in spring, no reference intervals were calculated for this season. Significant differences between the seasons of sampling were found for vitamins A (*p* = 0.0002), B1 (*p* < 0.0001), and B9 (*p* < 0.0001) (Table 1 and Table 2); significant sex-specific variations were only found for vitamin E (*p* = 0.0438) (Table 1). Significant interactions of season and sex with blood vitamin levels were found for vitamin B1 (*p* = 0.0148) (Table 2).

## 4. Discussion

Blood reference intervals for various vitamins are an important tool for monitoring the health of animals and to assure the provision of a sufficient supply through the diet, as well as to avoid over-supply. In the present study, reference intervals for vitamins A, B1, B2, B6, B9, B12, and E were established for pond sliders under managed care in Germany. Previously, reference intervals had only been established for a limited range of vitamins in a few reptile species. Vitamin A (0.5 ± 0.1 and 0.17 ± 0.08 mg/L) and E (8.0 ± 3.7 and 5.14 ± 5.26 mg/L) values reported in wild leatherback turtles (*Dermochelys coriacea*) [12,14] and adult loggerhead sea turtles (*Caretta caretta*) (vitamin A 0.51 ± 0.21 mg/L and E 4.39 ± 3.33 mg/L) [16] have varied depending on the study, and are similar to or slightly lower/higher than those found in pond sliders in the present study. This is not surprising, as the three species have a very different ecology and diets. Leatherback turtles and loggerhead sea turtles are found in tropical and subtropical regions of the oceans and feed predominantly on jellyfish [28] and benthic invertebrates and fish [16], respectively. Pond sliders, on the other hand, live in calm freshwater ponds which have a muddy, soft bottom and aquatic and terrestrial vegetation, so they feed mainly on aquatic plants, many invertebrates, and to a lesser extent vertebrates [18]. In comparison to Hermann’s tortoises (*Testudo hermanni*) (vitamin A 0.29 ± 0.14 mg/L; E 2.83 ± 2.17 mg/L) [15], vitamin A levels are lower and vitamin E levels are higher in pond sliders. This shows that it is important to establish species-specific reference intervals.

Currently, comparable values for vitamin B1, B2, B6, B9, and B12 have only been published for Hermann’s tortoises [15]. The values in Hermann’s tortoises [15] are all lower (B1 45.40 ± 21.67 µg/L; B2 486.88 ± 199.12 µg/L; B9 13.80 ± 6.44 ng/mL; B12 1020.37 ± 606.61 pg/mL), with the exception of B6 (9.64 ± 17.12 µg/L), which is higher in comparison to the measured values in pond sliders in the present study. Reasons for this are likely the differences in ecology and diets between the two species. Hermann’s tortoises are herbivorous terrestrial Mediterranean tortoises [29,30], with a natural habitat and diet that differs distinctly from that of pond sliders. There are several case reports on vitamin B1 deficiency in crocodilians and snakes associated with neurological disorders, but no blood vitamin levels were determined in these cases [3,4]. It is therefore still unclear at what level clinical signs of deficiency occur in which reptile species.

The present study found seasonal fluctuations in some vitamins, with vitamin A, B1, and B9 levels rising from spring to early summer and then decreasing in late summer. One reason for this could be the hibernation of the turtles, so the values are lowest in spring due to the long break in metabolism and feed intake, and the values in the blood rise again due to increasing intake with the diet. On the other hand, the feed spectrum and the vitamin content of the diet also fluctuate throughout the year, which can also lead to changes. For example, the animals eat more insects in summer and more plant-based feed in the fall [19]. The physiological form of the vitamins and their metabolism also play a role in this context. Fat-soluble vitamins, such as vitamins A and E, are stored in the liver and partly also in fatty tissue and released again when required so that a lower dietary intake can be compensated [1,31]. However, during hibernation, the liver metabolism is also reduced, which leads to a limited release of vitamin A from the liver tissue during this time. Water-soluble vitamins, such as B vitamins, on the other hand, are only stored in the body in very small amounts and an excess of many of these vitamins is usually excreted via the kidneys [1].

Vitamin E was the only vitamin for which significant differences were found between the sexes, with significantly higher values in the female turtles. This could be related to differences in metabolism between males and females, which is also influenced by hormones. However, vitamin E is also stored in fatty tissue [1,31], so females with more body fat may have more vitamin E stored, which may also mean more can be made available in the blood. It should also be noted that females mobilize more vitamins and minerals from their stores during egg development, and that vitamins are also incorporated into the egg [32].

In this study, blood samples from turtles from different keepers and kept under varying conditions were examined as part of clinical health checks. Feed intake was therefore not specifically monitored and the vitamin content of the feed was not measured. In addition, no tissue samples such as liver biopsies were examined, so no concrete statements can be made about the vitamin intake or stored vitamins. Only the vitamins currently circulating in the blood were determined.

## 5. Conclusions

This study establishes blood reference intervals for vitamins A, B1, B2, B6, B9, B12, and E in pond sliders for the first time. Vitamin A, B1, and B9 levels vary significantly between the seasons and vitamin E levels vary between the sexes. The established reference intervals serve as a basis for further studies, which should help to optimize the keeping and feeding of this species and to improve the medical treatment of deficiencies and intoxications.

## Figures and Tables

**Table 1:** Calculated reference intervals for fat-soluble vitamins in heparinized whole blood and plasma samples from pond sliders (*Trachemys* sp.); specific reference intervals for each season and sex are only listed if significant (*p* ≤ 0.05) differences were found.

Vitamin	Unit	Season/Sex	n	Mean	SD	Minimum	Maximum	Median	10% Percentile	90% Percentile	Lower RI (CI)	Upper RI (CI)	D	*p*-Value
A (retinol)	mg/L	All	188	0.20	0.12	0.03	0.65	0.17	0.08	0.34	0.1 (0.0–0.1)	0.5 (0.4–0.7)	NG	*p* < 0.001
		Spring	7	0.12	0.05	0.08	0.23	0.10	0.08	0.23	-	-	-	-
		Early summer	54	0.27	0.14	0.11	0.65	0.22	0.13	0.46	0.1 (0.1–0.1)	0.6 (0.6–0.7)	NG	*p* < 0.001
		Late summer	127	0.17	0.09	0.03	0.46	0.15	0.08	0.32	0.03 (0.0–0.1)	0.4 (0.3–0.5)	NG	*p* < 0.001
E (tocopherol)	mg/L	All	188	8.57	5.74	0.10	33.78	7.59	2.52	15.10	0.1 (0.1–1.8)	24.5 (19.1–33.8)	NG	*p* < 0.001
		Male	53	5.74	3.18	0.10	13.70	5.37	1.81	9.81	0.1 (0.1–1.1)	13.7 (10.3–13.7)	G	*p* = 0.351
		Female	135	9.68	6.14	0.10	33.78	9.03	3.38	17.14	0.5 (0.1–2.4)	26.7 (20.7–33.8)	NG	*p* < 0.001

CI 90%—confidence interval of the reference limits; D—distribution; G—Gaussian distribution; NG—non-Gaussian distribution; *p*-value of Anderson–Darling test for normal distribution threshold—*p* < 0.3.

**Table 2:** Calculated reference intervals for water-soluble vitamins in heparinized whole blood and plasma samples from pond sliders (*Trachemys* sp.); specific reference intervals for each season and sex are only listed if significant (*p* ≤ 0.05) differences were found.

Vitamin	Unit	Season/Sex	n	Mean	SD	Minimum	Maximum	Median	10% Percentile	90% Percentile	Lower RI (CI)	Upper RI (CI)	D	*p*-Value
B1 (thiamine pyrophosphate)	µg/L	All	163	63.21	33.79	0.50	225.00	59.10	26.40	102.30	9.7 (0.5–13.7)	137.1 (121.1–225.0)	NG	*p* < 0.001
		Spring	5	39.98	8.13	27.00	48.70	42.40	27.00	48.70	-	-	-	-
		Early summer	45	92.03	36.96	36.90	225.00	87.80	51.20	134.90	37.1 (36.9–50.1)	216.8 (163.7–225.0)	NG	*p* = 0.013
		Late summer	113	52.77	25.38	0.50	137.20	53.30	15.00	85.70	2.3 (0.5–11.9)	112.9 (96.9–137.2)	NG	*p* = 0.044
B2 (flavin adenine dinucleotide)	µg/L	All	148	747.06	407.38	73.00	2616.00	693.50	377.00	1026.00	238.5 (73.0–321.0)	2249.0 (1563.0–2616.0)	NG	*p* < 0.001
B6 (pyridoxal)	µg/L	All	160	4.05	6.49	0.20	64.49	2.37	0.78	8.01	0.2 (0.2–0.6)	19.4 (11.8–64.5)	NG	*p* < 0.001
B9 (folic acid)	ng/mL	All	164	16.09	11.14	0.60	80.35	16.06	4.51	23.82	2.3 (0.6–3.6)	50.9 (36.0–80.4)	NG	*p* < 0.001
		Spring	5	13.16	5.80	6.74	20.10	10.97	6.74	20.10	-	-	-	-
		Early summer	42	24.69	16.21	4.51	80.35	20.10	9.21	47.76	4.6 (4.5–7.0)	78.6 (53.5–80.4)	NG	*p* < 0.001
		Late summer	117	13.13	6.65	0.60	36.03	12.62	4.01	20.10	1.5 (0.6–2.9)	20.5 (20.1–36.0)	NG	*p* < 0.001
B12 (cobalamin)	pg/mL	All	165	3919.00	2500.18	472.10	10,001.00	3225.00	1287.00	6001.00	538.0 (472.1–1051.0)	10,001.0 (9275.0–10,001.0)	NG	*p* < 0.001

CI 90%—confidence interval of the reference limits; D—distribution; G—Gaussian distribution; NG—non-Gaussian distribution; *p*-value of Anderson–Darling test for normal distribution threshold—*p* < 0.3.

## Data Availability

The data that support the findings of this study are available from the corresponding author upon reasonable request.
